# Immune Checkpoint Inhibitors and Immune-Related Adverse Drug Reactions: Data From Italian Pharmacovigilance Database

**DOI:** 10.3389/fphar.2020.00830

**Published:** 2020-06-09

**Authors:** Rosanna Ruggiero, Federica Fraenza, Cristina Scavone, Gabriella di Mauro, Raffaele Piscitelli, Annamaria Mascolo, Carmen Ferrajolo, Concetta Rafaniello, Liberata Sportiello, Francesco Rossi, Annalisa Capuano

**Affiliations:** Campania Regional Centre for Pharmacovigilance and Pharmacoepidemiology, Department of Experimental Medicine, Section of Pharmacology “L. Donatelli”, University of Campania “Luigi Vanvitelli”, Naples, Italy

**Keywords:** immune-related ADRs, immune checkpoint inhibitors, pharmacovigilance, safety, spontaneous reporting system

## Abstract

**Background:**

The introduction of immune checkpoint inhibitors (ICIs) in clinical practice has brought significant benefits for patients. Seven ICIs are available in Europe: nivolumab, pembrolizumab, atezolizumab, avelumab, durvalumab, cemiplimab, and ipilimumab. Despite their proven clinical efficacy, these innovative drugs may cause serious immune-related adverse drugs reactions (irADRs). Given the significance of these ADRs for patients’ health, we analyzed individual case safety reports (ICSRs) related to ICIs, focusing on those reporting irADRs, collected in the Italian spontaneous reporting database.

**Methods:**

We analyzed ICI-induced irADRs collected in the Italian Pharmacovigilance database (Rete Nazionale di Farmacovigilanza [RNF]) from January 1, 2002, to February 28, 2019, focusing on those reported in the Campania Region. We retrieved from an open-access Italian pharmacovigilance system, the RAM system (for national safety data), and from the RNF (for Campania safety data) all ICSRs reporting ADRs related to ICIs authorized until the analysis date. Focusing on irADRs, we performed descriptive and disproportionality analyses through the reporting odds ratio (ROR) with 95% confidence interval.

**Results:**

*National results*. Among 2,088 ICI-related ICSRs, 801 reported irADRs. The majority of such ADRs occurred in male patients reporting gastrointestinal and skin toxicities. Nivolumab and pembrolizumab were drugs most commonly reported as suspect drugs. Compared to other ICIs, ROR was statistically significant for pembrolizumab and ipilimumab.

*Campania Region results*. Out of 253 ICI-related ICSRs sent to Regional Pharmacovigilance Center of Campania Region, 121 reported at least one ICI-induced irADR. These were serious in 37.2% of cases and had an unfavorable outcome in 32.2% of cases. Overall, out of 8 ICSRs reported ADR with a fatal outcome, four reported irADRs. From disproportionality analyses on Campania Region ICSRs, statistically significant ROR emerged only for ipilimumab.

**Conclusions:**

Our results showed that during the study period several serious irADRs were reported, some of which had fatal outcome. Given the clinical relevance of irADRs, further investigations in real-life context are necessary for a better characterization of ICIs safety profiles. Oncologists should be trained to early recognize and adequately manage irADRs. Patients should also be educated to immediately report any new symptom or worsening of pre-existed ones during the ICI treatment.

## Introduction

The introduction of immune checkpoint inhibitors (ICIs) in the oncological therapeutic arsenal has brought significant benefits for patients, leading to long-lasting tumor responses (Kadono, 2017). Therefore, these innovative drugs are increasingly used. ICIs are mainly represented by monoclonal antibodies able to target programmed cell death receptor or its ligand (PD-1/PD-L1) and cytotoxic T-lymphocyte-associated antigen 4 (CTLA-4). PD-1, PD-L1, and CTLA-4 are immune checkpoints, markedly overexpressed in the tumor microenvironment and involved in the inhibition of T cell signals (Teixidó et al., 2018). The expression of these immune checkpoints is one of several tumors adaptive responses to escape the immune system (La-Beck et al., 2015; Michot et al., 2016). Blocking these negative costimulatory molecules, ICIs re-establish the ability of cytotoxic T cells to destroy tumor cells. Acting on immune cells rather than cancer ones (Viggiano et al., 2012), ICIs have revolutionized the treatment of several types of cancer, leading to a substantial shift in oncology paradigms. Currently, seven ICIs are available in the European pharmaceutical market: the CTLA-4 inhibitor (CTLA-4i) ipilimumab (authorized in 2011); PD-1 inhibitors (PD-1i), nivolumab (2015), pembrolizumab (2015), and cemiplimab (June 2019); PD-L1 inhibitors (PD-L1i), atezolizumab (2017), avelumab (2017), and durvalumab (2018). Since the inhibition mediated by the association nivolumab/ipilimumab turns out better in improving anti-tumor responses in metastatic melanoma, the combination therapy was also authorized in May 2016 (Sosa et al., 2018). Despite their proven clinical efficacy, ICIs, as all monoclonal antibodies, are related to a new type of drug-toxicity (Brahmer et al., 2018), which includes immune-related ADRs (irADRs) (Koster et al., 2015). IrADRs might represent the consequence of the effects resulting from T cells acting against antigens shared by tumor and normal cells or from the concomitant activation of multiple T cell populations (Guan et al., 2015). On the other hand, their occurrence is strictly related to ICIs’ pharmacodynamic properties. Indeed, it is known that ICI-target immunosuppressive molecules are involved in self-tolerance as well in various autoimmune conditions (Brahmer et al., 2018). Acting on the immune system, ICI-induced irADRs can involve any tissue and organ and can occur anytime (Michot et al., 2016). However, differences in terms of types, rates, time to onset, and seriousness of irADRs have been described for CTLA-4i and PD-1/PD-L1i (Myers, 2018). Literature data highlighted that ICI-induced irADRs could occur more frequently than expected, such as in the case of gastrointestinal immune-related events, which are likely to be encountered more frequently by gastroenterologists, who will need to be aware of these ADRs. Therefore, the early recognition and treatment are very critical steps (Shivaji et al., 2019). Indeed, if not promptly recognized and properly managed (i.e., using corticosteroids or immunosuppressive drugs and/or suspending suspected drugs), irADRs can be life-threatening (Wang et al., 2018). Two recent pharmacovigilance studies have investigated the safety profile of ICIs, in terms of irADRs, using data from the international surveillance databases VigiBase. Among these, Salem et al. evaluated the association between ICIs and CV events. Their findings highlighted that ICIs were associated with higher reporting of myocarditis, pericardial diseases, and vasculitis. These CV-irADRs affected most commonly men and tended to occur within one month of the first ICI administration(Salem et al., 2018). Johnson et al. evaluated instead neurologic ADRs in patients receiving ICIs. The results revealed that ICIs were associated with higher incidence of myasthenia gravis (ROR, 16.5; 95% confidence interval [95% CI], 14.5–18.9; IC025, 3.31), encephalitis (ROR, 10.4; 95% CI, 9.2–11.8; IC025, 3.15), and meningitis (ROR, 3.1; 95% CI, 2.5–3.9; IC025, 1.01). Most of these ADRs were considered as irADRs (Shivaji et al., 2019). Therefore, many studies have already investigated the occurrence of specific subtypes of irADRs related to ICIs, using data from post-marketing surveillance database, but to our knowledge no studies have investigated the reporting of all ICI-induced irADRs in the Italian context.

Given the clinical relevance of ICI-induced irADRs and considering that underlying mechanisms are still not completely understood (Postow et al., 2018), as for any other innovative drug, extensive safety monitoring is highly recommended (Cimmaruta et al., 2016). Therefore, in order to extrapolate as much information as possible from the context of clinical practice (Scavone et al., 2017c), we analyzed individual case safety reports (ICSRs) related to ICIs, collected into the National Pharmacovigilance Network (Rete Nazionale di Farmacovigilanza, RNF) focusing on those reporting irADRs, with a specific analysis in the Campania Region (South of Italy).

## Materials and Methods

### Data Source

In Italy, pharmacovigilance activities are coordinated by the Italian Medicine Agency (AIFA), which instituted in 2001 the RNF, a national pharmacovigilance database. The RNF allows the collection, management, and analysis of ICSRs reporting ADRs, defined by Directive 2010/84/EU, occurring throughout the national territory (Sessa et al., 2015). In Italy, Pharmacovigilance Regional Centers (PRCs) contribute to the evaluation of the quality of collected data and the causality assessment for each drug or vaccine/ADR couple cooperating with AIFA to vaccines and drug safety signal detection. Each PRC can access to ICSRs collected in the RNF that refer to ADRs occurred in its competence territory, on which it may conduct further analysis such as preventability of reported ADRs (Sessa et al., 2017; Mascolo et al., 2019). Moreover, starting from July 2017, the AIFA has set up an online open-access system (report Reazioni Avverse dei Medicinali, RAM), which allows access to some data relating to ICSRs recorded into the RNF starting from 2002. Data available from the RAM system can contribute to obtaining an overview of ADRs that occur at a national level. Therefore, we retrieved from the RAM system (for national safety data) and the RNF (for Campania safety data) all ICSRs reported from January 1, 2001, to February 28, 2019, in which pembrolizumab, nivolumab, ipilimumab, atezolizumab, avelumab, or durvalumab were indicated as suspected drug. Cemiplimab was not considered in our analysis given its recent authorization.

### Descriptive and Disproportionality Analyses

First, we carried out a descriptive analysis of all national ICI-related ICSRs in terms of number of ICSRs, seriousness, gender, age groups, System Organ Class (SOC), and p-term of irADRs (**Table 1 Supplementary Materials**) both for the entire ICIs class and single active substance. Moreover, we performed a descriptive analysis of ICI-related ICSRs reported in the Campania Region, focusing on those reporting irADRs. These regional ICSRs were stratified by suspected drugs, concomitant drug(s), therapeutic indication, median age (IQR), gender, seriousness (serious or non-serious/non-defined), time to event (TTE), time to resolution of event(s) (TTR), outcome, causality assessment, and irADR(s) management. According to current pharmacovigilance regulations, all ADRs that induced death, hospitalization or prolongation of hospitalization, severe or permanent disability, life threat, congenital abnormalities/birth deficits or considered as clinically relevant were categorized as serious. The outcome was categorized as favorable if irADR was completely resolved or improved, while as unfavorable if irADR resolved with sequelae, was unchanged or induced patient’s death. The causality assessment was performed through the Naranjo algorithm (Naranjo et al., 1981). We used the chi square test, the Mann-Whitney U test, or the Fisher exact test (where appropriate) to evaluate if differences were statistically significant (*p* < 0.05). Finally, we performed a disproportionality analysis of national and regional ICI-induced irADRs cases, through the reporting odds ratio (ROR) with 95% CI, using other ICIs as comparison. The signal was considered statistically significant when the lower limit of 95% CI of a ROR exceeded 1.0.

## Results

### National Results

**Figure 1:**
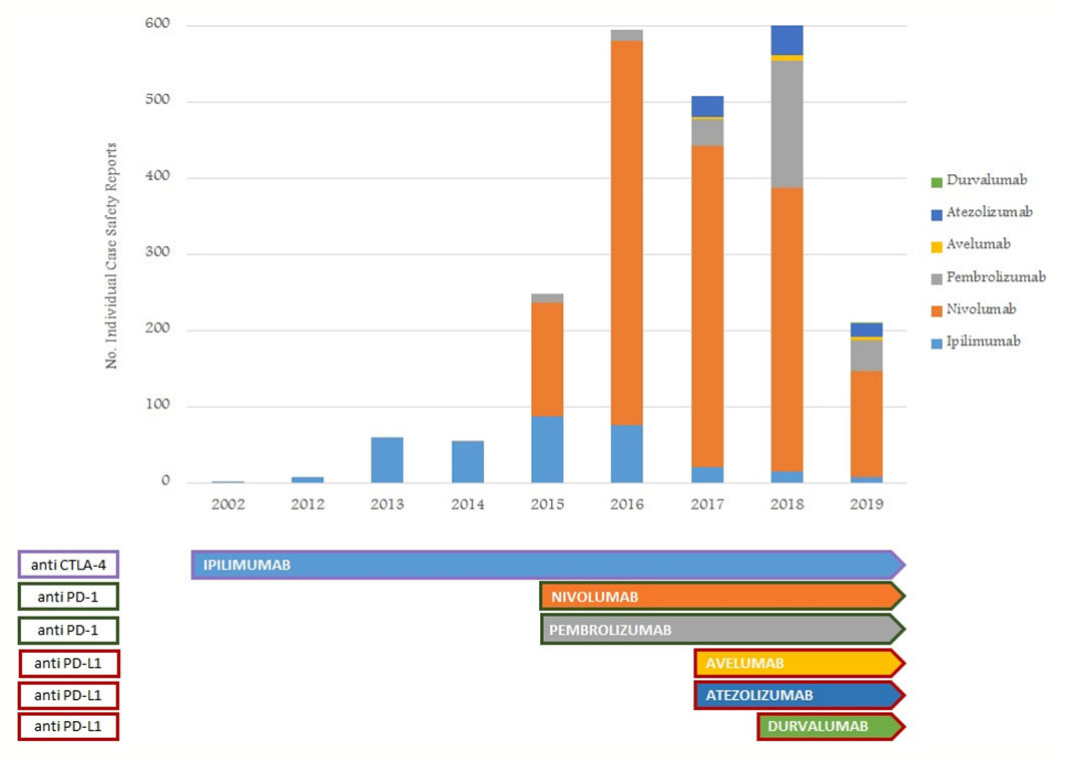
Trend of number of ICI-related ICSRs from January 1, 2002, to February 28, 2019, collected into the RNF and listed in the RAM system.

From January 1, 2002, to February 28, 2019, the reporting of ICI-induced ADRs progressively increased; this growth was more evident for nivolumab-related ICSRs since 2015 (**Figure 1**). ICSRs related to ipilimumab before the authorization date may represent those collected in the context of clinical trials or compassionate use programs. Overall 2088 ICSRs with an ICI as suspected drug were collected in the RNF and listed in the RAM system. About 70% of these ICSRs were related to nivolumab (n = 1,452), followed by ipilimumab (n = 318; 15%), pembrolizumab (n = 230; 11%), atezolizumab (n = 78; 4%), and avelumab (n = 9; < 1%). Only 1 ICSR reported durvalumab as suspected drug. Demographic characteristics and seriousness classification of national ICSRs stratified for each ICI are shown in **Table 1**. No substantial difference emerged in terms of seriousness for any single drug. The majority of ICSRs reported ADRs that occurred in male patients (> 58% for each single ICI) and in the age group > 66 years, except for ipilimumab, for which ADRs were more frequently reported in the age group 18 to 65 years. Gender differences were statistically significant for nivolumab (p < 0.05), ipilimumab (p < 0.001), and atezolizumab (p < 0.05) (data not shown). In the analysis of distribution of ICI-induced ADRs for SOCs, we found a greater involvement of “General disorders and administration site conditions” (n = 558; 14%), “Respiratory, thoracic, and mediastinal disorders” (n = 485; 12%), and “Gastrointestinal disorders” (n = 481; 12%), followed by “Skin and subcutaneous tissue disorders” (n = 414; 10%), “Investigations” (n = 354; 9%), and “Musculoskeletal and connective tissue disorders” (n = 195; 5%) (data not shown).

**Table 1:** ICI-related ICSRs collected into the RNF from January 1, 2002, to February 28, 2019 stratified by seriousness, gender, and age groups (data available from the RAM system).

ICI as suspect drug	Tot. ICSRs	Seriousness			Gender			Age Groups			
		Serious	Not Serious	N.A.	M	F	N.A.	12-17 years	18–65 years	≥66 years	N.A.
**Nivolumab**	1452	695	635	122	958	430	64	4	540	708	200
**Ipilimumab**	318	157	150	11	185	129	4	1	174	128	15
**Pembrolizumab**	230	131	98	1	152	74	4	–	87	121	22
**Atezolizumab**	78	38	38	2	61	16	1	–	17	42	19
**Avelumab**	9	5	4	–	6	2	1	–	1	7	1
**Durvalumab**	1	1	–	–	–	1	–	–	1	–	–

N.A, Not Available.

#### National irADRs Results

**Figure 2:**
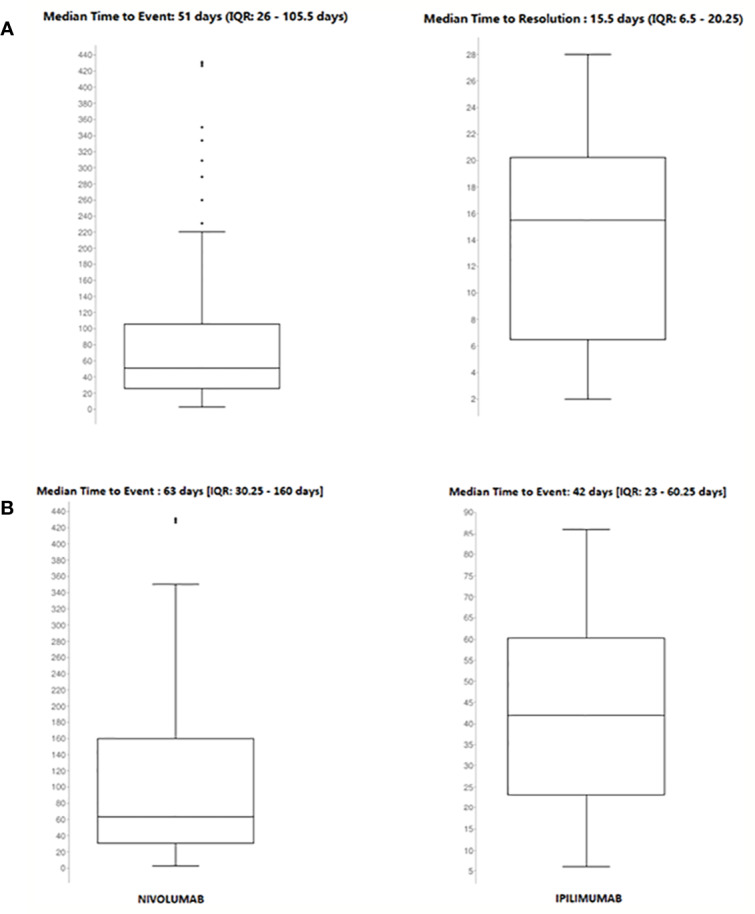
Disproportionality analyses of the national **(A)** and regional data **(B)**.

Among 2088 ICSRs, we found that 20% of all ADRs were immune-related (801/3988). Majority of these irADRs were signs or symptoms of gastrointestinal toxicity (33%; mainly represented by diarrhea, pancreatitis, and enterocolitis), followed by skin toxicity (17%; mainly itch, psoriasis, and macular-papular rash) and pulmonary disorders, such as pneumonia and pleurisy (16%) (**Table 2**). Moreover, several irADRs involved also the hematologic and endocrine systems. While pulmonary ADRs were mainly induced by both nivolumab and ipilimumab (59/129 and 57/129, respectively), most hematologic irADRs were related to nivolumab (81/98). IrADRs involving cardiovascular and renal systems were less frequently reported. Nivolumab was the ICI most commonly reported as suspected drug (n = 478), followed by ipilimumab (n = 192) and pembrolizumab (n = 106). A statistically significant ROR was found for ipilimumab and pembrolizumab (**Figure 2A**). According to the results of disproportionality analysis, a statistically significant ROR was found for ipilimumab (ROR, 2.9050; 95% CI, 2.2733–3.7122) and pembrolizumab (ROR, 1.4305; 95% CI, 1.0857–1.8847) (**Figure 2A**). Therefore, ipilimumab and pembrolizumab were associated with an increased reporting probability of irADRs rather than no-irADRs if compared to other ICIs.

**Table 2:** ICI-induced irADRs reported from January 1, 2002, and February 28. 2019 and collected into the RNF (data available from the RAM system).

TOXICITY TYPE	*Tot. irADRs* *n (%)*	*ICIs*					
		Nivolumab n (%)	Pembrolizumab n (%)	Atezolizumab n (%)	Ipilimumab n (%)	Avelumab n (%)	Durvalumab n (%)
**Gastrointestinal Toxicity**	264 (33)	132 (50)	22 (8)	2 (1)	108 (41)	-	-
Diarrhea	225	115	11	2	97	–	–
Pancreatitis	20	12	6	–	2	–	–
Enterocolitis	12	5	5	–	2	–	–
Celiac Disease	3	–	–	–	3	–	–
Gastritis	2	–	–	–	2	–	–
Ileitis	2	–	–	–	2	–	–
**Skin Toxicity**	139 (17)	103 (74)	22 (16)	8 (6)	6 (4)	-	-
Itch	94	71	16	6	1	–	–
Psoriasis	20	15	2	2	1	–	–
MP Rash	13	10	2	–	1	–	–
SJS	8	4	2	–	2	–	–
Vitiligo	4	3	–	–	1	–	–
**Pulmonary Toxicity**	129 (16)	59 (46)	11 (8)	1 (1)	57 (44)	-	1 (1)
Pneumonia	87	59	11	1	15	–	1
Pleurisy	42	–	–	–	42	–	–
**Hematological Toxicity**	98 (12)	81 (83)	8 (8)	9 (9)	–	–	–
Hemolytic Anemia	41	37	–	4	–	–	–
Thrombocytopenia	37	31	2	4	–	–	–
Neutropenia	20	13	6	1	–	–	–
**Endocrine Toxicity**	91 (11)	52 (57)	17 (19)	1 (1)	21 (23)	-	-
Hyperthyroidism	36	32	–	1	3	–	–
Hypothyroidism	18	5	10	–	3	–	–
Hypophysitis	16	7	5	–	4	–	–
Adrenal insufficiency	16	7	2	–	7	–	–
Diabetes	5	1	–	–	4	–	–
**Liver Toxicity**	22 (2.7)	11 (50)	10 (45)	1 (5)	-	-	-
Hepatitis	22	11	10	1	–	–	–
**Musculoskeletal Toxicity**	19 (2.4)	14 (74)	4 (21)	1 (5)	-	-	-
Arthritis	11	9	1	1	–	–	–
Myositis	7	5	2	–	–	–	–
Myopathies	1	–	1	–	–	–	–
**Central Toxicity**	16 (2)	8 (50)	7 (44)	-	-	1 (6)	-
Neuropathy	7	4	3	–	–	–	–
Encephalitis	6	4	2	–	–	–	–
GBS	2	–	2	–	–	–	–
Myasthenia	1	–	–	–	–	1	–
**Ocular Toxicity**	9 (1.1)	8 (89)	1 (11)	-	-	-	-
Conjunctivitis	6	6	–	–	–	–	–
Uveitis	3	2	1	–	–	–	–
**Renal Toxicity**	7 (0.9)	5 (71)	2 (29)	-	-	-	-
Nephritis	7	5	2	–	–	–	–
**Cardiovascular Toxicity**	7 (0.9)	5 (71)	2 (29)	-	-	-	-
Myocarditis	4	3	1	–	–	–	–
Pericarditis	2	1	1	–	–	–	–
Vasculitis	1	1	–	–	–	–	–

MP rash, maculo-papular rash; SJS, Steven Johnson’s syndrome; GBS, Guillain-Barré syndrome.

### Campania Region Results

In the same study period, 253 ICSRs related to ICIs were sent to the Campania PRC. All ICSRs were spontaneous reports, except for 4 ICSRs collected within a non-interventional multiregional study (MEREAFAPS study) (data not shown) (Perrone et al., 2014). The majority of ICSRs reported not-serious ADRs (N = 176; 69%) (**Table 2 Supplementary Materials**). Nivolumab was most commonly reported suspected drug (N = 172; 68%), followed by ipilimumab (N = 45; 17.8%), and pembrolizumab (N = 33; 13%). There were only three ICSRs related to atezolizumab and none related to avelumab, durvalumab, or the combination therapy. Serious ADRs were reported in almost 30% of all ICSRs for each ICI, except for pembrolizumab, which had the lowest percentage of serious ADRs (15.2%). Demographic and clinical characteristics of overall cases are shown in **Table 3**. ICI-induced ADRs occurred in patients with a median age of 66 years (IQR, 57–72 years) and no difference were found for irADRs, likewise no-irADRs occurred in patients with a median age of 65.5 years (IQR, 57–71.5 years). Both immune- and non-immune-related ADRs occurred mainly in male patients, with a slightly higher percentage of no-irADRs compared to irADRs in males (65.9% vs. 54.5%); no statistically significant difference emerged in terms of gender (p > 0.05). The majority of ICSRs reported ADRs that were classified as not serious (69.6%). The percentage of not serious ADRs was higher for no-irADRs compared to irADRs (78.8% vs. 59.5%, respectively), while the opposite was found for serious ADRs (19.7% vs. 37.2%, respectively). Comparing the seriousness degree for irADRs and no-irADRs, a statistical significant difference was found (p < 0.05). Among ICSRs reporting serious ADRs, the most common seriousness criteria was “clinical relevant” both for irADRs and no-irADRs (26.4% vs. 15.2%). Differences were not statistically significant. The majority of ICSRs reported ADRs with a favorable outcome (60.9%), and no statistically significant differences were found between ICSRs reporting irADRs and no-irADRs. Among ICSRs reporting unfavorable outcomes (N = 67; 26.5%), eight cases (seven related to nivolumab and one to ipilimumab) resulted in patient’s death (**Table 4**), mainly due to ADRs involving respiratory and cardiac systems. In 2 cases (cases n. 4 and 5), the reporter specified that patient’s death was considered as related to the patient’s clinical conditions.

**Table 3:** Demographic and clinical characteristics of ICI-related irADRs cases and no-irADRs cases sent through the Campania Region spontaneous reporting system from January 2001 to February 2019.

Variable	Level	All cases, N = 253 (100%)	irADR cases, n = 121 (47.8%)	No-irADRs cases, n = 132 (52.2%)	p-value (< 0.05)
**Age**	Median (IQR)	66 (57–72)	66 (58–73)	65.5 (57–71.5)	>0.05*
	Missing	9	5	4	
**Gender**					>0.05**
	Male	153 (60.5)	66 (54.5)	87 (65.9)	
	Female	94 (37.1)	49 (40.5)	45 (34.1)	
	Missing	6 (2.4)	6 (5)	–	
**Seriousness of adverse events**					0.001978
	Not serious	176 (69.6)	72 (59.5)	104 (78.8)	
	Serious	71 (28)	45 (37.2)	26 (19.7)	
	NA	6 (2.4)	4 (3.3)	2 (1.5)	
**Seriousness criteria**					>0.05
	Clinically relevant	52	32 (26.4)	20 (15.2)	
	Hospitalization or its prolongation	15	11 (9)	4 (3)	
	Death	3	1 (0.8)	2 (1)	
	Life-threating	1	1 (0.8)	–	
**Outcome**					>0.05
	Favorable (completely resolved or improved)	154 (60.9)	71 (58.7)	83 (62.9)	
	Unfavorable (death, unchanged or resolved with sequelae)	67 (26.5)	39 (32.2)	49 (37.1)	
	Not available	32 (12.6)	11 (9.1)	21 (15.9)	
**Suspected drugs**	Nivolumab	172 (68)	74 (61.2)	98 (74.3)	0.025845
	Ipilimumab	45 (17.8)	34 (28.1)	11 (8.3)	0.00004
	Pembrolizumab	33 (13)	12 (9.9)	21 (15.9)	>0.05
	Atezolizumab	3 (1.2)	1 (0.8)	2 (1.5)	>0.05

* Value of the Mann-Whitney U Test.

** Value of the Fisher exacts test statistic.

**Table 4:** ICSRs sent to the Campania Pharmacovigilance Regional Centre from January 2001 to February 2019 reporting immune-related adverse drug reactions resulted in patient’s death.

Case n.	Age (years)	Sex	Suspected drugs	Ther. Indic.	Concomitant drugs	ADR(s)	TTE (days)	TTD (days)	Causality Assessment
**1**.	78	F	Ipilimumab	Mel. M.	–	Diarrhea, Thrombocytopenia	65	~3	Possible
**2**.	72	M	Nivolumab	L.C.	–	Autoimmune hepatitis	3	9	Possible
**3**.	57	F	Nivolumab	L.C.	Zoledronic Acid	Dyspnea, Peripheral edema	10	6	Possible
**4**.	66	F	Nivolumab	L.C.	Metformin Levothyroxine Alprazolam Paroxetine Trazodone	Respiratory failure	25	1	Possible
**5**.	50	M	Nivolumab	L.C.	–	Cardiac arrest	18	18	Possible
**6**.	70	M	Nivolumab	Mel.	–	Pneumonia, death	N.A.	N.A.	Possible
**7**.	66	F	Nivolumab	L.C.	Levothyroxine	AV block, eyelid ptosis, hypophysitis, myasthenia, diarrhea, and pneumonia	~21	~210	Possible
**8**.	67	M	Nivolumab	L.C.	Acetylsalicylic acid	Hemoptysis	15	7	Possible

Mel. M., metastatic melanoma; L.C., lung cancer; Mel., melanoma; AV block, atrioventricular block.

#### Campania Region irADRs Results

One-hundred-twenty-one out of 253 ICSRs sent to the Campania PRC reported an ICI-induced irADRs (47.8%), which were serious in 37.2% of cases (including several 3–4 grade cases) and had unfavorable outcome in 32.2% of cases. Four irADRs were fatal (cases n. 1, 2, 6, and 7 reported in **Table 4**). Nivolumab was the ICI most commonly involved in the occurrence of irADRs (N = 74) (**Table 3**), but the majority of these had favorable outcomes. Statistically significant differences in terms of irADR/no-irADRs cases emerged for both nivolumab and ipilimumab (p < 0.05). Looking at each single ICI, the majority of ipilimumab-induced ADRs were irADRs (75.5%), with unfavorable outcomes in most of the cases (61.8%). Pembrolizumab-induced irADRs were those with the most commonly favorable outcome (75%). Moreover, more than 33% of irADRs reported for each ICI were serious (**Table 3 Supplementary Materials**). The majority of irADRs involved skin disorders (mainly represented by itch and psoriasis nivolumab-induced), followed by gastrointestinal toxicity (mainly ipilimumab-induced diarrhea) (**Table 5**). Several cases of irADRs involved the endocrine system, such as hypothyroidism and hypophisitis, which were mainly induced by nivolumab. Moreover, nivolumab was the ICI most commonly involved in several cases of pneumonia and thrombocytopenia. The causality assessment was possible for most cases. Concomitant diseases were reported in 19% of cases, and those most commonly reported were cardiovascular (i.e., hypertension, myocardial infarction, atrial fibrillation), and endocrine disorders (i.e., diabetes mellitus 2, hypothyroidism, multinodular goiter). We found a case of nivolumab-induced pancreatic toxicity occurred in a 71 years old patient with HCV-related hepatocarcinoma. Only 15 ICSRs reported concomitant drugs (≥3 concomitant drugs per ICSR), mainly represented by cardiovascular and lipid-lowering drugs.

**Table 5:** ICI-induced immune-related adverse drug reactions reported in the Campania Region and collected into the RNF.

TOXICITY TYPEs	irADRs N=132	ICIs			
		Nivolumab	Ipilimumab	Pembrolizumab	Atezolizumab
		N=85	N=34	N=12	N=1
**Skin Toxicity**	49 (37.1%)	30 (35.3%)	12 (35.3%)	7 (58.3%)	–
Itch	32	15	12	5	
Psoriasis	6	6	–	–	
Macular-papular rash	4	4	–	–	
Vitiligo	3	3	–	–	
Epidermolysis bullosa	2	–	–	2	
Pemphigus	2	2	–	–	
**Gastrointestinal toxicity**	32 (24.2%)	14 (16.5%)	17 (50%)	1 (8.3%)	–
Diarrhea	24	9	14	1	
Enterocolitis	4	1	3	–	
Pancreatitis	4	4	–	–	
**Endocrine toxicity**	22 (16.7%)	19 (22.4%)	–	2 (16.7%)	1 (100%)
Hypothyroidism	13	11		2	–
Hypophysitis	3	3		–	–
Thyroiditis	3	3		–	–
Hyperthyroidism	2	2		–	–
Thyrotoxicosis	1	–		–	1
**Pulmonary toxicity**	9 (6.8%)	6 (7.1%)	1 (2.9%)	2 (16.7%)	–
Pneumonia	8	6	1	1	
Respiratory failure	1	–	–	1	
**Hematological toxicity**	7 (%)	5 (5.8%)	2 (5.9%)	–	–
Thrombocytopenia	6	5	1		
Neutropenia	1	–	1		
**Liver toxicity**	5 (3.8%)	3 (3.5%)	2 (5.9%)	–	–
Hepatitis	5	3	2		
**Musculoskeletal toxicity**	3 (2.3%)	3 (3.5%)			–
Myositis	2	2			
Arthritis	1	1			
**Central toxicity**	3 (2.3%)	3 (3.5%)	–	–	–
Neuropathy	1	1			
Encephalitis	1	1			
Myasthenia	1	1			
**Ocular toxicity**	1 (0.8%)	1 (1.2%)	–	–	–
Uveitis	1	1			
**Renal toxicity**	1 (0.8%)	1 (1.2%)	–	–	–
Nephritis	1	1			

**Figure 3:**
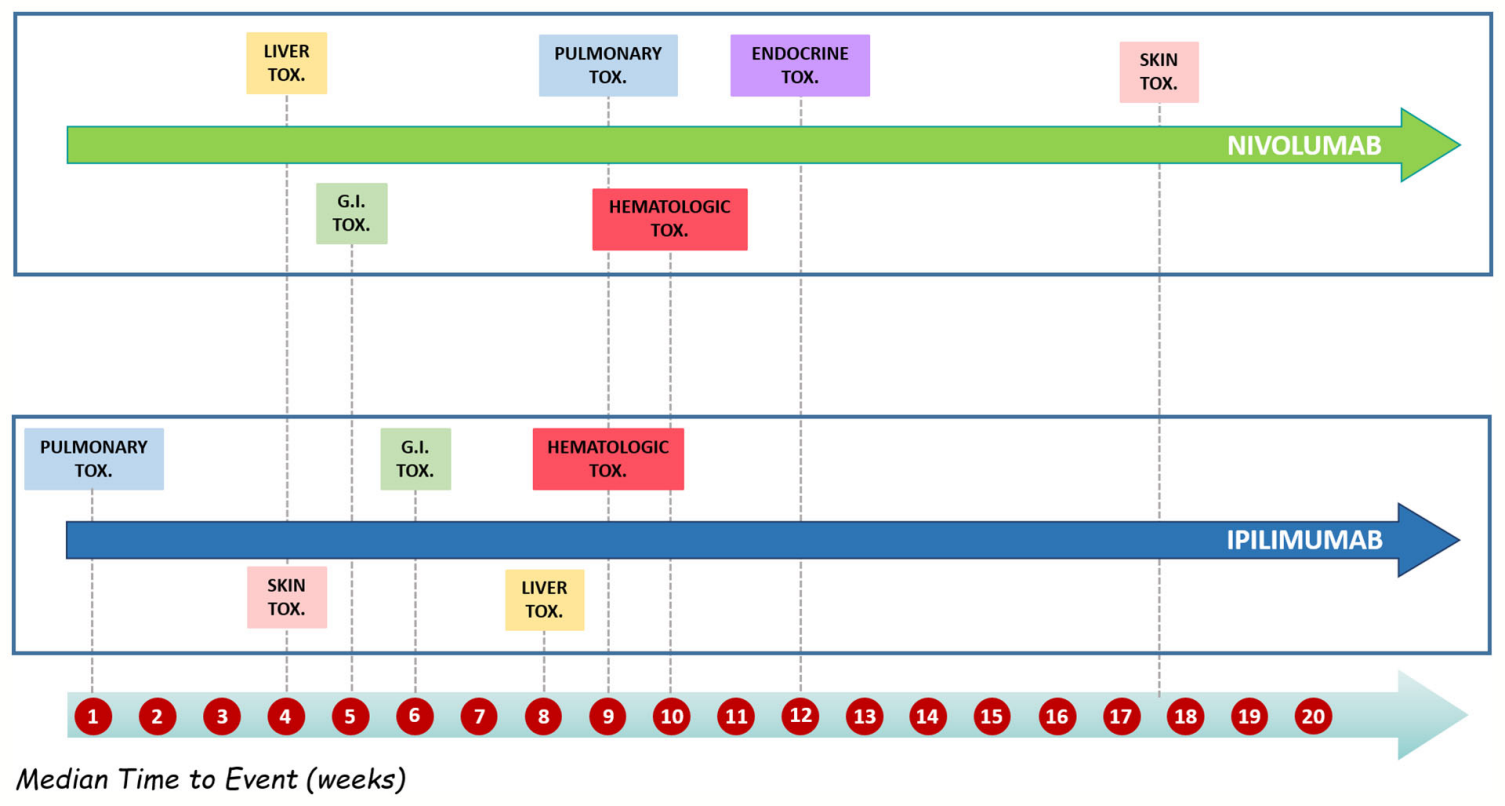
**(A)** Median time to event and median time to resolution of ICI-induced irADRs reported in the Campania Region and collected into the RNF from January 1, 2001, to February 28, 2019. **(B)** Comparing median time to event of nivolumab- and ipilimumab-induced irADRs reported in Campania Region and collected in RNF from January 1, 2001, to February 28, 2019.

**Figure 4:**
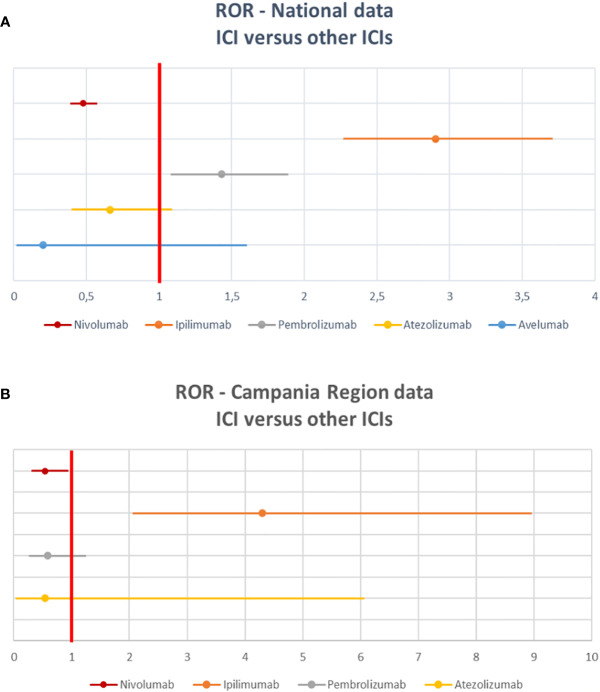
Distribution of median time to event of ipilimumab- and nivolumab-induced irADRs reported in the Campania Region and collected into the RNF from January 1, 2001, to February 28, 2019 stratified by toxicity types.

Details of meaningful reported ICI-induced cases were described in **Table 4 of Supplementary Materials**. The reporter specified the autoimmune etiology of the ADR in 6 ICSRs, reporting cases of hepatitis (N = 3), encephalitis (N = 1), hypothyroidism (N = 1), and pancreatitis (N = 1). In most cases, irADRs were managed with ICI discontinuation and the administration of high-dose corticosteroids. The corticosteroids administration allowed favorable outcomes in 75% of cases in which they were administered. Lastly, among many serious irADRs involving the gastrointestinal system, we found 2 cases of ipilimumab-induced diarrhea, which required the administration of infliximab to manage the ADR. Infliximab administration allowed the improvement of ADR in one of these cases. The median TTE was 51 days (IQR, 26–105.5), and the median TTR was 15.5 days (IQR, 6.5–20.25) (**Figure 3A**) for the entire ICI class. Comparing TTE related to nivolumab and ipilimumab, which were ICIs most frequently reported, we found that ipilimumab-induced events occurred earlier compared to nivolumab, with median TTE of 42 days (IQR, 23–60.25) and 63 days (IQR, 30.25–160), respectively (**Figure 3B**). All ipilimumab-induced irADRs occurred within 9 weeks. Hepatic, gastrointestinal, and skin toxicity occurred earlier. Nivolumab-induced endocrine and skin irADRs occurred later (**Figure 4**). According to the results of disproportionality analysis, ipilimumab was associated with an increased reporting probability of irADRs rather than no-irADRs if compared to other ICIs. In fact, a statistically significant ROR emerged only for ipilimumab (**Figure 2B**). Finally, excluding ipilimumab-induced irADRs cases and comparing only PD-L1i and PD-1i cases, a statistically significant ROR was not found (ROR, 1.3699; 95% CI, 0.8528–2.2006) (data not shown).

## Discussion

Our study aimed to analyze ICI-induced irADRs collected in the Italian pharmacovigilance database, with a focus on the Campania Region. To our knowledge, this study represents the first comprehensive evaluation of safety data related to ICIs using the Italian spontaneous reporting system. During ICIs treatment irADRs is quite common, with an incidence of 90% for any-grade irADRs, even though in most cases these are mild and transient (Puzanov et al., 2017). In our study, irADRs represented 20% of all ICI-related ADRs collected into the RNF. In the Campania Region almost half of ICI-related ICSRs reported irADRs. Differences in terms of safety have been highlighted among different ICIs classes. IrADRs incidence seems to be higher with ipilimumab than nivolumab and pembrolizumab (Buchbinder and Desai, 2016; Megiorni et al., 2017; Varricchi et al., 2017). It has been hypothesized that the CTLA-4 inhibition induces a larger T-cell proliferation, while PD-1 blockade leads to an activation of a smaller number of T-cell clones. Slight differences in toxicity profiles of PD-1i and PD-L1i have also been found, but to date clinical data are limited (Varricchi et al., 2017). In line with literature data, in our study ipilimumab treatment appeared unwieldy compared to other ICIs, especially for the onset of serious irADRs with unfavorable outcomes. This seems to be confirmed also by significant statistically ROR emerged for both national and regional data. Statistically significant ROR emerged also for pembrolizumab from disproportionality analysis of national data, showing that also this treatment could increase the risk of reporting irADRs compared to other ICIs. On the contrary, although nivolumab was the ICI reported as suspected drug in the highest number of ICSRs, it was not associated with an increased reporting probability of irADRs compared to no-irADR when compared to the other ICIs, resulting in a safe and widely used drug. Due to their better safety profiles, PD-1i and PD-L1i are more commonly used than CTLA-4i (Myers, 2018). Furthermore, nivolumab showed equivalent efficacy profile compared to ipilimumab (Weber et al., 2017). This evidence could justify the larger number of ICSRs related to nivolumab.

We have found statistically significant gender-differences in our national analysis. Specifically, nivolumab-, ipilimumab-, and atezolizumab-induced ADRs were more commonly reported in males compared to females. Sex-based immunological differences, expressed in different immune responses to infections as well as in different predisposition to develop autoimmune diseases are reported in literature (Taneja, 2018). However, the gender-influence on the efficacy and safety of immunotherapy treatments seems to be controversial (D’Amici et al., 2013; Conforti et al., 2018; Grassadonia et al., 2018; Wallis et al., 2019). What we have found should be related to gender differences in incidence rates of diseases representing therapeutic indications of ICIs treatment. For example, highest lung cancer and melanoma incidence rates have been recognized in men (Vavalà et al., 2016; Smalley, 2018). Furthermore, our result should be related to the hypothesized but still debated (Postow et al., 2018) correlation between irADRs onset and immunotherapy efficacy (Haratani et al., 2018; Sato et al., 2018), the latter seems to be superior in men (Conforti et al., 2018; Grassadonia et al., 2018; Wu et al., 2018). Since ICIs act on the immune system, irADRs can involve any tissue and organ, probably due to activation of autoreactive T cells damaging host tissues (Sanchez et al., 2019). As confirmed from our analysis, the majority of irADRs expressed mainly in cutaneous and gastrointestinal toxicity (Myers, 2018). We found two cases, both induced by ipilimumab, of severe diarrhea that required infliximab administration. According to the ESMO guidelines, infliximab treatment is necessary when high corticosteroids administration have been not sufficient to manage the irADR (Haanen et al., 2017; Haanen et al., 2018). Rare irADRs, like renal, cardiac, and ocular ADRs, have been reported, confirming that such ADRs occurred less frequently (Puzanov et al., 2017). From the national data set, beyond irADR involving skin and gut, we found several cases of pulmonary and hematologic toxicities, although generally these systems are involved less often in irADRs (Varricchi et al., 2017). On the other hand, in the regional dataset, we found several cases of irADRs involving the endocrine system, such as hypothyroidism and thyroiditis, mainly due to nivolumab-treatment. According to literature data, it seems that thyroiditis is more common with PD-1i (Puzanov et al., 2017). In this respect, cases reported in our study were all nivolumab-induced. Regarding timing of onset, except for a slight delay of nivolumab-induced skin toxicity, median TTEs are consistent with the timing emerged during the clinical studies, for both nivolumab and ipilimumab (Varricchi et al., 2017). Events mainly occurred within the first 3 months from the starting of the treatment. Moreover, our results confirmed that ipilimumab-induced ADRs occur earlier than ones induced by nivolumab. This could be related to different functions of CTLA-4 and PD-1 in the immune response (Buchbinder and Desai, 2016). CTLA-4 is involved in proximal steps, while PD-1 takes part in later stages of the immune response (Weber et al., 2012; Varricchi et al., 2017).

According to Champiat et al., both concomitant diseases and drugs could promote the onset of irADRs (Champiat et al., 2016). In our results, among drugs already related to autoimmune toxicity and so considered such as predisposing drugs to irADRs (Chang and Gershwin, 2010), antihypertensive (such as atenolol) and lipid-lowering drugs (such as atorvastatin) were those most commonly reported. This could be related to the ability of atenolol to induce the formation of anti-histone antibodies, which are more expressed in patients affected by autoimmune diseases (Gouet et al., 1986; McGuiness et al., 1997). In the same way, also statins seem to be able to modulate the immune system through different mechanisms, inducing possible development of autoimmune diseases (Chapman-Shimshoni et al., 2003). Other possible risk factors for irADRs seem to be concomitant diseases. From our analysis, we found several cases of thyroid disorders and one of HCV-hepatitis. HCV infection has been associated to various immune and autoimmune disorders. In case of failure in the virus eradication process, a persistent infection could lead to excessive immune stimulation through a humoral and cell-mediated response, whose dysfunction has been associated with the increase of immune complexes and autoantibodies, responsible for immune disorders (McMurray and Elbourne, 1997; Paroli et al., 2012). A recent meta-analysis showed that most of CTLA-4 deaths occurred in patients who experienced colitis, while fatalities related to PD-1/PD-L1i occurred in patients who experienced pneumonitis, hepatitis, and neurotoxic effects (Brahmer et al., 2018). In line with this, autoimmune hepatitis and pulmonary disorders were reported in 4 out of 6 fatal cases related to nivolumab. The only one fatal case induced by ipilimumab was due to severe thrombocytopenia. Although hematologic ADRs rarely occur during ICIs treatment, thrombocytopenia could be caused by autoantibodies that bind platelet epitope, such as glycoproteins, interfering with their function (Stasi and Provan, 2004; Provan and Newland, 2015). Among our fatal cases, a nivolumab-related cardiac arrest has been reported. Cardiac arrest could be the result of acute myocarditis, a well-known ICI-related cardiac irADR (Ammirati et al., 2017). According to Johnson et al the presence of an antigen shared between cancer cells and myocardiocytes may explain ICI-induced myocarditis. Whilst the activated T cells are able to provide an anti-tumor response binding to the antigen expressed on tumor cells, they could induce inflammation and necrosis of myocardiocytes, by penetrating the myocardium (Johnson et al., 2016). Generally, cardiac irADRs are less common.

## Strengths and Limitations

Although the spontaneous reporting system is a cornerstone of pharmacovigilance activities, it is characterized by intrinsic limitations such as the under-reporting, that is the failure to report a suspected ADR by medical or healthcare personnel, and the possible incorrect or incomplete information reported in the ICSR. Given these limitations and considering that ICI-prescription were not available, we were not able to define the real incidence of ICI-induced irADRs neither to establish which ICI was associated with the greatest number of irADRs.

Despite these limitations, it is largely recognized that the spontaneous reporting system represents a simple, useful, and inexpensive pharmacovigilance tool. Analysis of spontaneous reporting data contributes to a better characterization of the drug safety profiles, especially for newly authorized medicines. It allows detecting rare and serious ADRs detectable only in the post-marketing phase when a larger population compared to those in clinical trials use drugs. Furthermore, the spontaneous reporting system involves ICSRs related to frail population, including elderlies and patients with multiple comorbidities, that are usually excluded by the premarketing clinical trials; therefore, data obtained from the spontaneous reporting system represent one of the main source of information for these population. Lastly, considering the recent authorization of all ICIs, the peculiarity of their toxicity profiles and given the lack of safety data from the real life context, our study represents the first comprehensive evaluation of safety data related to ICIs, using the Italian spontaneous reporting system.

## Conclusions

From our analysis emerged that several serious irADRs have been reported, some of which with fatal outcome. Overall, 2088 ICSRs related to ICIs were collected in the RNF. Twenty percent of these ICSRs reported irADRs. Gastrointestinal and skin toxicities were the most common reported irADRs. In the same study period, 253 ICI-related ICSRs were reported to the Campania PRC. Almost half of regional ICSRs reported ICI-induced irADRs, which were serious in 37.2% of cases and had an unfavorable outcome in 32.2% of cases. Overall, we found 8 fatal cases. Although nivolumab was the ICI most commonly involved in the occurrence of irADRs, most serious cases were ipilimumab-induced, involving gut. Rare irADRs, like those involving renal, cardiac, and ocular systems, have been also reported. Statistically significant differences between cases of irADR and no-irADR emerged for nivolumab and ipilimumab (p < 0.05). Ipilimumab-induced events occurred earlier compared to nivolumab-induced ones.

Given the clinical relevance of irADRs and considering that innovative drugs are increasingly authorized by accelerated procedures (Scavone et al., 2017b; Scavone et al., 2017a), further safety investigations in real-life context are necessary (Scavone et al., 2019). We believe that our results could be shared with clinicians that dailies prescribe and administer ICIs or monoclonal antibodies in general. The importance of these innovative drugs in improving patients’ clinical conditions is undeniable, but it should be noted that ADRs still represent one of the leading causes of death among inpatients, especially those belonging to frail population or those with multiple comorbidities. Therefore, it is desirable that safety data from a real-life context, such as those presenting in this paper, could serve as source of new information for drugs recently authorized, like many of ICIs nowadays available, and hopefully as a reminder for a systematic and careful monitoring of patients receiving ICIs. Based on our results and in line with other literature data, we believe that oncologists should pay attention to concomitant drugs and diseases, by strictly monitoring those patients who present such risk factors for irADRs onset (Cole et al., 2019; Martins et al., 2019). Patients should be educated to immediately report any new or worsening symptoms (Wood et al., 2019). It is essential to intervene when the severity of irADRs is still slight, avoiding interruption of lifesaving immunotherapeutic treatments.

## Data Availability Statement

The datasets generated for this study will not be made publicly available: National dataset is available online, while the access to the regional dataset requires the approval of the Italian Medicine Agency.

## Ethics Statement

Ethical review and approval was not required for the study on human participants in accordance with the local legislation and institutional requirements. Written informed consent from the participants’ legal guardian/next of kin was not required to participate in this study in accordance with the national legislation and the institutional requirements.

## Author Contributions

RR and FF wrote the manuscript. CS, FR, and AC made critical revisions to the manuscript. CF, CR, and LS designed the research. RR, FF, GM, RP, and AM performed the research. RR, FF, CR, CS, and AC analyzed the data. FR and AC developed the concept; all authors approved the final version of the manuscript.

## Conflict of Interest

The authors declare that the research was conducted in the absence of any commercial or financial relationships that could be construed as a potential conflict of interest.
