# Supplementary material for: Immune Checkpoint Inhibitors and Immune-Related Adverse Drug Reactions: Data From Italian Pharmacovigilance Database
Source: Front Pharmacol. 2020 Jun 9;11:830. doi: 10.3389/fphar.2020.00830 (PMC7295943; doi:10.3389/fphar.2020.00830)
Supplement: Supplementary file 1 [file DataSheet_1.docx]

Supplementary Material

**Immune checkpoint inhibitors and immune-related adverse drug reactions: data from Italian pharmacovigilance database.**

Rosanna Ruggiero^1^* & Federica Fraenza^1^*; Cristina Scavone^1^; Gabriella di Mauro^1^; Raffaele Piscitelli,^1^; Annamaria Mascolo^1^; Carmen Ferrajolo^1^; Francesco Rossi^1^; Concetta Rafaniello^1^; Liberata Sportiello^1^; Annalisa Capuano^1^.

1 Campania Regional Centre for Pharmacovigilance and Pharmacoepidemiology - Department of Experimental Medicine – Section of Pharmacology “L. Donatelli”, University of Campania “Luigi Vanvitelli”, Naples (Italy)

* these authors have equally contributed.

**Corresponding author:**

Cristina Scavone
cristina.scavone@unicampania.it

**Keywords:** immune-related adverse drugs reactions, immune checkpoint inhibitors, pharmacovigilance, safety.

**Supplementary Table 1. Immune-related adverse events examined in ICIs-related ICSRs.**

| ***toxicity types*** | **p-term** |
| --- | --- |
|  |  |
| ***Skin Toxicity*** | Muscular-papular rash  Itch  Psoriasis  Vitiligo  DRESS / Stevens Johnson Syndrome  Lyell syndrome |
| ***Gastrointestinal toxicity*** | Enterocolitis  Diarrhea  Pancreatitis  Gastritis  Ileitis  Celiac disease |
| ***Endocrine toxicity*** | Hyperthyroidism  Hypothyroidism  Hypophysitis  Diabetes  Adrenal insufficiency |
| ***Pulmonary toxicity*** | Pneumonia  Pleurisy  Sarcoid granulomatosis |
| ***Ocular toxicity*** | Uveitis  Conjunctivitis  Scleritis / episcleritis Blepharitis  Retinitis  Choroiditis  Orbital myositis |
| ***Musculoskeletal toxicity*** | Myositis  Myopathies  Arthritis  Dermatomyositis |
| ***Cardiovascular toxicity*** | Myocarditis  Pericarditis  Vasculitis |
| ***Liver toxicity*** | Hepatitis |
| ***Renal toxicity*** | Nephritis |
| ***Central toxicity*** | Neuropathy  Myelopathy  Guillain-Barrè syndrome  Encephalitis / Meningitis  Myasthenia |
| ***Hematological toxicity*** | Hemolytic anemia  Thrombocytopenia  Neutropenia  Hemophilia  Pancytopenia |

**Supplementary Table 2. ICSRs reporting ICIs-induced ADRs sent through the Campania Region spontaneous reporting system from January 2001 to February 2019 and collected into the RNF stratified for seriousness and single suspected drug.**

| **Suspect drug** | **TOT**  **N=253 (100%)** | **Serious**  **N=71 (28%)** | **Not-Serious**  **N=176 (69.6%)** | **N.A.**  **N=6(2.4%)** |
| --- | --- | --- | --- | --- |
| **Nivolumab** | 172 (68%) | 50 (29) | 117 (68) | 5 (2.9) |
| **Ipilimumab** | 45 (17.8) | 15 (33.3) | 29 (64.5) | 1 (2.2) |
| **Pembrolizumab** | 33 (13%) | 5 (15.2) | 28 (84.8) | - |
| **Atezolizumab** | 3 (1.2%) | 1 (33.3) | 2 (66.7) | - |

**Supplementary Table 3. Immune-related adverse drug reactions stratified for each suspected drug by seriousness and outcome.**

|  | | **SERIOUSNESS** | | **OUTCOME** | | |
| --- | --- | --- | --- | --- | --- | --- |
| **Suspect drug** | **ICSRs reporting irADRs**  **N (%)** | **ICSRs reporting serious irADRs**  **N (%)** | **ICSRs reporting not serious irADRs**  **N (%)** | **Favorable**  **N (%)** | **Unfavorable**  **N (%)** | **N.A.**  **N (%)** |
| **Nivolumab N=172** | **74 (43)** | **28 (37.8)** | **46 (62.2)** | **49 (66.2)** | **17 (23)** | **8 (10.8)** |
| **Ipilimumab N=45** | **34 (75.5)** | **12 (35.3)** | **22 (64.7)** | **12 (35.3)** | **21 (61.8)** | **1 (2.9)** |
| **Pembrolizumab N=33** | **12 (36.4)** | **4 (33.3)** | **8 (66.7)** | **9 (75)** | **2 (16.7)** | **1 (8.3)** |
| **Atezolizumab**  **N=3** | **1 (33.3)** | **1 (100)** | **-** | **1 (100)** | **-** | **-** |
| **TOT = 253** | **121** | **45** | **76** | **71** | **40** | **10** |

**Supplementary Table 4. Individual Case Safety Reports related to irADRs cases having at least one ICI as suspected drug and sent through the Campania Region spontaneous reporting system from January 2001 to Febraury 2019.**

| **CASES OF ATEZOLIZUMAB-INDUCED irADRs** | | | | | | | | | |
| --- | --- | --- | --- | --- | --- | --- | --- | --- | --- |
| ***Case n.*** | ***Sex*** | ***Age*** | ***Ther. Indic.*** | ***TTE (days)*** | ***p-term*** | ***Causality assessment*** | ***Concomitant drug(s)*** | ***irADR management*** | ***Outcome*** |
|  | F | 65 | L.C. | 19 | Thyrotoxicosis | Possible | - | Thiamazole administration,  unmodified ICI dose | Favor. - I |
| **CASES OF IPILIMUMAB-INDUCED irADRs** | | | | | | | | | |
| ***Case n.*** | ***Sex*** | ***Age*** | ***Therapeutic indication*** | ***TTE (days)*** | ***p-term*** | ***Causality assessment*** | ***Concomitant drug(s)*** | ***irADR management*** | ***Outcome*** |
|  | F | 38 | Mel. | 7 | Diarrhea G3 | Probable | Dexamethasone | Corticosteroids administration,  ICI discontinuation | Unfavor.-RwS |
|  | M | 41 | Mel. | 41 | Hyperbilirubinemia G4 | Possible | - | ICI discontinuation | Unfavor.-U |
|  | F | 70 | Mel. | 61 | Neutropenia G3 | Possible | - | ICI discontinuation | NA |
|  | F | 43 | Mel. | 42 | Hypertransaminasemia G3 | Probable | - | ICI discontinuation | Favor. - R |
|  | M | 87 | Mel. | 41 | Diarrhea G2 | Possible | - | ICI discontinuation | Favor. - R |
|  | F | 44 | Mel. | 43 | Diarrhea G3 | Probable | - | Infliximab administration,  ICI discontinuation | Favor. - I |
|  | F | 36 | Mel. | 85 | Diarrhea G4 | Possible | - | Corticosteroids administration, Unmodified ICI dose | Unfavor.-RwS |
|  | F | 68 | Mel. | 16 | Diarrhea G4 | Possible | - | Infliximab administration,  ICI discontinuation | Unfavor.-U |
|  | M | 67 | Mel. | 6 | Pneumonia | Possible | - | Corticosteroids administration, Unmodified ICI dose | Favor. - R |
|  | F | 77 | Mel. | 44 | Diarrhea G3 | Possible | - | Corticosteroids administration, Unmodified ICI dose | Favor. - R |
|  | F | 77 | Mel. | 65 | Diarrhea Thrombocytopenia | Possible | - | ICI discontinuation, Corticosteroids administration,  transfusion of plates | Unfavor.-D |
|  | F | 71 | Mel. | NA | Diarrhea | Possible | - | NA | Unfavor.-RwS |
|  | M | 52 | Mel. | 63 | Hepatotoxicity G4 | Possible | - | Corticosteroids administration,  Unmodified ICI dose | Unfavor.-U |
|  | M | 46 | Mel. | 46 | Autoimmune hepatitis | Probable | - | Corticosteroids administration | NA |
|  | M | 64 | Mel. |  | Hypertransaminasemia G4 | Possible | - | ICI discontinuation | NA |
| **CASES OF PEMBROLIZUMAB-INDUCED irADRs** | | | | | | | | | |
| ***Case n.*** | ***Sex*** | ***Age*** | ***Therapeutic indication*** | ***TTE (days)*** | ***p-term*** | ***Causality assessment*** | ***Concomitant drugs*** | ***irADR management*** | ***Outcome*** |
|  | M | 71 | Mel. | 11 | Chest pain, Cough, Respiratory failure | Possible | ***-*** | Corticosteroids administration,  ICI discontinuation | Favor. - I |
|  | M | 72 | L.C. | 3 | Pneumonia | Possible | ***-*** | ICI discontinuation | Favor. - R |
|  | M | 76 | L.C. | 12 | Epidermolysis bullosa G3 | Possible | ***-*** | Corticosteroids administration,  ICI discontinuation | ***NA*** |
|  | M | 76 | L.C. | 260 | Epidermolysis bullosa | Possible | ***-*** | ***NA*** | Favor. - I |
|  | M | 54 | L.C. | 1 | Hyperpyrexia | Possible | ***-*** | Corticosteroids administration | Favor. – I |
| **MEANINGFUL CASES OF NIVOLUMAB-INDUCED irADRs** | | | | | | | | | |
| ***Case n.*** | ***Sex*** | ***Age*** | ***Therapeutic indication*** | ***TTE (days)*** | ***p-term*** | ***Causality assessment*** | ***Concomitant drugs*** | ***irADR management*** | ***Outcome*** |
|  | M | 66 | L.C | 47 | Cough,  Shortness of breath, Interstitial pneumonia | Possible | Omeprazole, Bisoprolol, Ticlopidine, Tramadole, Pregabalin,  Alfuzosine, Glycopyrronium bromide | Corticosteroids administration  Unmodified ICI dose | Unfavor.-U |
|  | M | 60 | L.C | 140 | Interstitial pneumonia | Probable | Pantoprazole  Prednisone, Cetirizine | Unmodified ICI dose | NA |
|  | - | 75 | Mel. | 156 | Hypothyroidism | Probable | - | NA | Favor. – I |
|  | F | 71 | L.C | 63 | Pneumonia  G3 | Possible | - | Oxygen therapy  Corticosteroids administration  ICI discontinuation | Unfavor.-U |
|  | M | 80 | Mel. | 28 | Autoimmune myositis, Neuropathy G3 | Possible | - | Corticosteroids administration  ICI discontinuation | Favor. – I |
|  | M | 71 | L.C | - | Pancreatic toxicity | Possible | Atenolol Nicardipine Esomeprazole | ICI discontinuation | Favor. – I |
|  | - | 63 | L.C | 50 | Pancreatitis | Possible | - | Corticosteroids administration | Favor. – I |
|  | M | 16 | Hodg.L | 31 | Autoimmune encephalitis | Possible | - | - | Favor. – R |
|  | F | 66 | L.C | 8 | Dyspnea, Pneumonia, Fever, Iatrogenic immuno-related pneumonia | Probable | Levothyroxine | Corticosteroids administration | Favor. – R |
|  | F | 66 | - | 31 | Autoimmune hepatitis Increased transaminases  Asthenia | Possible | - | Corticosteroids administration  ICI discontinuation | Favor. – I |
|  | F | - | L.C | - | AV block  Eyelid ptosis Hypothyroidism  Myasthenia gravis-like syndrome, Pneumonia Hypophysitis | Possible | Levothyroxine | Piridostigmine and Corticosteroids administration | Unfavor.-U |
|  | M | - | L.C | 61 | Glomerulonephritis, Hypophysitis | Possible | - | ICI discontinuation | - |
|  | M | 72 | Cancer NOS | 3 | Autoimmune hepatitis | Possible | - | - | Unfavor.-D |
|  | M | 62 | L.C | 57 | Dyspnea,  Interstitial pneumonia, Pleural effusion | Possible | - | Corticosteroids and antibiotics administration  Oxygen therapy | Favor. – I |
|  | F | 75 | L.C | 63 | Autoimmune hypothyroidism | Possible | - | Replacement therapy | Unfavor.-RwS |
|  | M | 78 | L.C | 35 | Abdominal pain, Confusion, Autoimmune pancreatitis | Possible | Omeprazole, Acetaminophen, darbepoetin alpha , Oxycodone/nalo-xone , Calcitriol, Tamsulosin hydrochloride | Corticosteroids administration | Favor. – R |
|  | F | 45 | L.C | 126 | Uveitis | Possible | - | Corticosteroids administration | Favor. – I |
|  | M | 58 | L.C | 142 | Myositis | Possible | - | - | Favor. – I |
|  | F | 63 | L.C | 95 | Itching, iatrogenic thyroiditis | Possible | - | Levothyroxine administration  Unmodified ICI dose | Favor. – I |
|  | F | 63 | L.C | 89 | Iatrogenic thyroiditis | Possible | - | Levothyroxine administration  Unmodified ICI dose | Favor. – I |

AV block= Atrioventricular block; Favor-I = improvement; Favor-R. = resolved completly; Hodg.L = Hodgkin Lymphoma; LC = Lung Cancer; Mel. = Melanoma; NA = not available; NOS = not otherwise specified; R.C = Renal Cancer; Unfavor.-D = death; Unfavor.-RwS = resolved with sequaele; Unfavor.-U = unchanged.
